# Neonatal respiratory morbidity following exposure to chorioamnionitis

**DOI:** 10.1186/s12887-017-0878-9

**Published:** 2017-05-17

**Authors:** Amy Metcalfe, Sarka Lisonkova, Yasser Sabr, Amelie Stritzke, KS Joseph

**Affiliations:** 1 Department of Obstetrics and Gynecology, University of Calgary, Foothills Medical Centre, 4th Floor North Tower, 1403 29th St NW, Calgary, AB T2N 2T9 Canada; 2 Department of Obstetrics and Gynecology, University of British Columbia, Vancouver, BC Canada; 3 Department of Obstetrics and Gynecology, King Saud University and King Khalid University Hospital, Riyadh, Saudi Arabia; 4 Department of Pediatrics, Section of Neonatology, University of Calgary, Calgary, AB Canada; 5 School of Population and Public Health, University of British Columbia, Vancouver, BC Canada

**Keywords:** Chorioamnionitis, Bronchopulmonary dysplasia, Respiratory distress syndrome, Fetuses at risk

## Abstract

**Background:**

There are conflicting results in the literature on the impact of chorioamnionitis on neonatal respiratory morbidities. However, most studies are based on small clinical samples and fail to account for the competing risk of perinatal death. This study aimed to determine whether chorioamnionitis affects the incidence of respiratory distress syndrome (RDS) and bronchopulmonary dysplasia (BPD) after accounting for the increased risk of death.

**Methods:**

Retrospective cohort study using linked birth and infant death registration and hospitalization records from Washington State between 2002 and 2011 (*n* = 763,671 singleton infants and *n* = 56,537 singleton preterm infants). Logistic regression models based on the traditional and fetuses-at-risk approaches were used to model two composite outcomes namely RDS and perinatal death and BPD and perinatal death. Confounders adjusted for in the models included maternal age, race, diabetes, hypertension, antenatal corticosteroids, mode of delivery and infant sex.

**Results:**

While models using the traditional approach found a significant association only between chorioamnionitis and composite BPD and perinatal death (OR = 1.23, 95% CI: 1.01–1.50); using the fetuses-at-risk approach, there was a significant association between chorioamnionitis and both composite outcomes (RDS and perinatal death OR = 2.74, 2.50–3.01; BPD and perinatal death OR = 5.18, 95% CI: 4.39–6.11).

**Conclusion:**

The fetuses-at-risk approach models the causal impact of chorioamnionitis on the development of the fetal lung and shows an increased risk of RDS, BPD and perinatal death associated with such maternal infection.

## Background

The impact of chorioamnionitis on fetal lung development and subsequent neonatal respiratory outcomes is controversial. A 2009 narrative review of the impact of histological chorioamnionitis on respiratory outcomes in premature infants found heterogeneous results [1]. Four (of 10) studies showed a decreased incidence of respiratory distress syndrome (RDS) following fetal exposure to histological chorioamnionitis, while 6 (of 10) studies found no difference [1]. Six (of 18) studies found an increased risk of bronchopulmonary dysplasia (BPD) following fetal exposure to histological chorioamnionitis, 1 (of 18) study found a decreased risk, and 11 (of 18) studies found no effect [1]. However, a 2012 systematic review and meta-analysis of 59 studies concluded that chorioamnionitis was a significant risk factor for BPD [2]. Despite differing conclusions about the role of chorioamnionitis on neonatal respiratory outcomes, the authors of both studies commented on substantial between-study heterogeneity related to the definitions of chorioamnionitis (clinical vs. histological) and BPD [1, 2]. There were also important differences in management of RDS, inclusion criteria and failure to adjust for confounding variables such as gestational age and exposure to antenatal steroids, as well as evidence of publication bias [1–3].

Some authors have proposed an ‘early protection, late-damage’ mechanism of action whereby antenatal inflammation promotes lung maturation in the premature infant, thus reducing the risk of RDS, and simultaneously causing an acute injury and leaving the lung vulnerable to additional post-natal injuries (such as those caused by mechanical ventilation and infection), and thereby increasing the risk of BPD [4–7]. The apparently protective effect of chorioamnionitis on RDS was first documented by Watterberg and colleagues based on a study of 53 mechanically ventilated infants admitted to NICU in the late 1980s [8]. However, the relevance of this study given changing practices in neonatology has been questioned [1]. None of the infants in the Watterberg study received antenatal corticosteroids or surfactant – two practices that are commonplace today. Furthermore, changes to gentler means of ventilation (such as continuous positive airway pressure (CPAP)) may also help reduce further insults to the developing lung [1]. Additionally, the synthesis of epidemiological findings in both the 2009 and 2012 reviews did not unanimously support this hypothesis, and postmortem examinations of stillbirths affected by chorioamnionitis and infants who died from RDS are similar, suggesting that chorioamnionitis does not protect the fetal lung [9]. Understanding of the role of chorioamnionitis in neonatal respiratory morbidities is also complicated because stillborn fetuses and live born infants who die during the neonatal period are typically excluded from studies examining the impact of chorioamnionitis on respiratory outcomes. This is problematic as chorioamnionitis has been shown to increase the risk of neonatal death [10]. While clinical chorioamnionitis affects between 16 and 66% of premature babies born at <34 weeks of gestation, its causative role in stillbirth is estimated to play a role in only about a quarter of cases in developed countries [11, 12]; thus the systematic exclusion of these infants induces selection bias. To our knowledge, only one study has examined the impact of chorioamnionitis on the composite outcome of BPD or death and found a significant association (OR = 2.73, 95% CI: 1.00–7.42) [4], and no studies have used the composite outcome of RDS or death. This study aimed to determine whether chorioamnionitis affects the incidence of neonatal respiratory outcomes such as respiratory distress syndrome and bronchopulmonary dysplasia (after accounting for effects on perinatal death).

## Methods

This study used individual-level de-identified routinely collected data on births (Birth Events Records Database (BERD)), hospitalizations (Comprehensive Hospital Abstract Records Database (CHARS)) and infant deaths in Washington State, USA. Singleton births (livebirths and stillbirths) without congenital anomalies (International Classification of Disease, 9th version clinical modification (ICD-9-CM) codes 740–759) that occurred between January 1, 2002 and December 31, 2011 were identified in BERD and CHARS data. The study focus was restricted to singleton preterm births with a recorded gestational age between 22 and 36 weeks since RDS and BPD are diseases of prematurity (*n* = 56,537 singleton preterm infants). This restricted population of interest was analyzed within the context of the full cohort of births that occurred between 22 and 42 weeks of gestation (*n* = 763,671 singleton infants) in order to estimate outcome rates under the fetuses-at-risk formulation (see below). An exemption for ethical review was granted by the Washington State Institutional Review Board as this study was considered research involving routine, publicly available, anonymized data.

Women with chorioamnionitis were identified by reviewing their hospitalization records for the ICD-9-CM code 658.4 (infection of amniotic cavity), their infant’s hospitalization records for the ICD-9-CM code 762.7 (fetus or newborn affected by complications of placenta, cord and membranes: chorioamnionitis), or documentation of chorioamnionitis on the birth certificate. This case definition refers to clinical chorioamnionitis in the assessment of the clinician treating the mother and/or infant, as rates and results of pathological placental investigations could not be determined from these data sources [13]. The definition of clinical chorioamnionitis is subject of ongoing debate and is variably diagnosed by presence of the following criteria: maternal intrapartum fever, maternal and/or fetal tachycardia, purulent amniotic or vaginal fluids, uterine tenderness and maternal leukocytosis [14]. BPD was identified using ICD-9-CM code 770.7 (chronic respiratory disease arising in the perinatal period) in the infant’s hospitalization records, while RDS was identified using ICD-9-CM code 769 (respiratory distress syndrome). While the database does not give further diagnostic detail, the definition of BPD followed the Eunice Kennedy Shriver National Institute of Child Health and Human Development consensus definition [15]. The clinical definition of RDS was presumably based on the requirement for surfactant.

Two methodological issues were addressed in this analysis in order to provide insight into the conflicting findings in the literature. First, potential confounding of the chorioamnionitis-respiratory outcome relation by gestational age was addressed using two alternative formulations, namely, the traditional perinatal formulation (with the denominator for gestational age-specific rates being total births) and the fetuses-at-risk formulation (denominator for gestational age-specific rates being fetuses in-utero). For instance, the gestational-age-specific outcome rates at 22 weeks were calculated using total births at 22 weeks at the denominator, whereas all live births and stillbirths at 22 weeks and beyond were used as the denominator for the calculation of gestational-age-specific outcome rates under the fetuses-at-risk formulation. Secondly, associations between chorioamnionitis and RDS, and chorioamnionitis and BPD were re-evaluated with the outcomes revised to encompass the composite outcomes of RDS and perinatal death (stillbirth or neonatal death), and BPD and perinatal death.

Using both the traditional and fetuses-at-risk formulations, descriptive statistics were used to characterize the population and logistic regression was used to evaluate the association between chorioamnionitis and the composite outcomes of RDS/perinatal death and BPD/perinatal death following adjustment for potential confounders. Potential confounders included race, maternal age, diabetes, hypertension, fetal sex, antenatal steroids and mode of delivery. Under the traditional approach, logistic regression analysis included gestational age as a covariate, while under the fetuses-at-risk approach, gestational age at birth was considered a factor on the causal pathway between chorioamnionitis and the outcomes, and therefore not included in the regression model. All analyses were conducted using Stata SE version 14 (College Station, Texas).

## Results

**Fig. 1:**
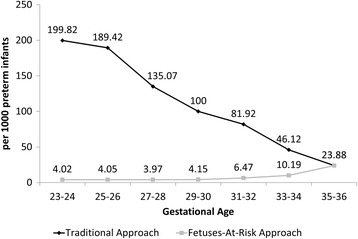
Incidence rate of chorioamnionitis by gestational age. Washington State Preterm Births 2002–2011 *n* = 56,537

Overall, 2631 out of 56,537 (4.65%, 95% CI: 4.48–4.83) pregnancies resulting in a preterm birth were complicated by chorioamnionitis. Under the fetuses-at-risk approach, the incidence rate of chorioamnionitis was stable until 30 weeks of gestation, at which point it increased in a linear fashion; whereas, under the traditional approach, the incidence rate of chorioamnionitis peaked at 23–24 weeks and declined steadily until 36 weeks of gestation (Fig. 1). Significant differences were also observed in the socio-demographic and clinical characteristics of pregnancies affected by chorioamnionitis (Table 1). Women with chorioamnionitis were significantly more likely to also have preterm premature rupture of membranes (*p* < 0.001), receive antenatal corticosteroids (*p* < 0.001), and have their infant admitted to a neonatal intensive care unit (*p* < 0.001), while their infants had lower gestational ages (*p* < 0.001) and were more likely ventilated (*p* < 0.001) (Table 1).

**Table 1:** Population characteristics – Washington State preterm births 2002–2011 *n* = 56,537

Variable	Mother did not have chorioamnionitis % (95% CI)	Mother had chorioamnionitis % (95% CI)	*p*-value
Maternal Race			<0.001
Non-hispanic white	56.8 (53.4–57.2)	54.4 (52.5–56.3)	
Non-hispanic black	4.3 (4.2–4.5)	6.5 (5.6–7.5)	
Hispanic	17.5 (17.2–17.8)	15.8 (14.5–17.3)	
Other/unknown	21.4 (21.0–21.7)	23.2 (21.6–24.8)	
Infant Sex			
Male	54.2 (53.7–54.6)	52.7 (50.7–54.7)	
Female	45.8 (45.4–46.2)	47.3 (45.3–49.3)	0.16
Preterm premature rupture of membranes	19.7 (19.4–20.0)	45.8 (43.9–47.7)	<0.001
Small for gestational age (<10th percentile)	13.4 (13.1–13.7)	7.1 (6.2–8.2)	<0.001
Antenatal steroids	1.0 (0.9–1.0)	19.2 (17.6–20.9)	<0.001
Admission to neonatal intensive care unit	36.9 (36.4–37.3)	59.8 (57.7–61.8)	<0.001
Mechanical ventilation of the neonate	7.6 (7.3–7.8)	14.6 (13.3–16.0)	<0.001
Respiratory distress syndrome	11.3 (11.1–11.6)	25.1 (23.5–26.8)	<0.001
Bronchopulmonary dysplasia	1.5 (1.4–1.6)	7.7 (6.8–8.8)	<0.001
Stillbirth	3.2 (3.1–3.4)	4.7 (4.0–5.6)	<0.001
Infant Death	0.4 (0.3–0.4)	0.3 (0.2–0.7)	0.80
	Mean (Standard Deviation)	Mean (Standard Deviation)	
Maternal age (years)	28.0 (6.3)	27.4 (6.4)	<0.001
Gestational age at delivery (weeks)	34.1 (2.9)	31.1 (4.5)	<0.001
Birth weight (grams)	2587.2 (1185.3)	2086.9 (1447.9)	<0.001

**Fig. 2:**
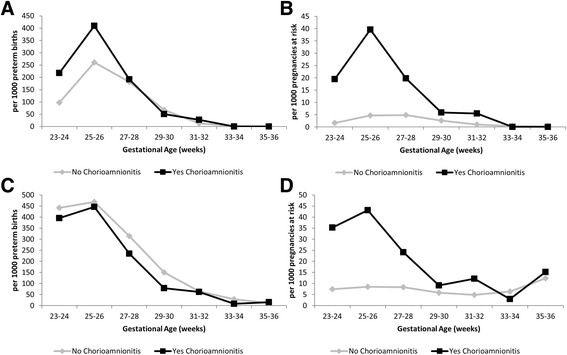
Incidence of bronchopulmonary dysplasia (Panels **a** & **b**) and composite bronchopulmonary dysplasia and perinatal mortality (Panels **c** & **d**) using the traditional (Panels **a** & **c**) and fetuses-at-risk (Panels **b** & **d**) approach. Washington State Preterm Births 2002–2011 *n* = 56,537

**Fig. 3:**
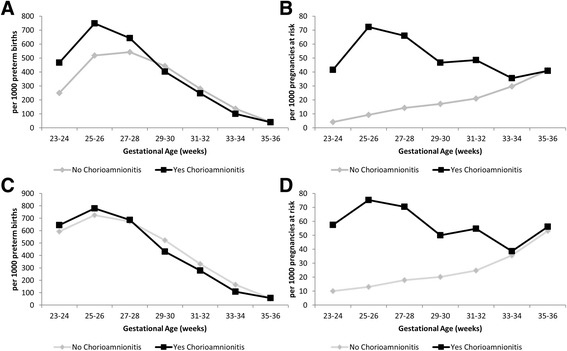
Incidence of respiratory distress syndrome (Panels **a** & **b**) and composite respiratory distress syndrome and perinatal mortality (Panels **c** & **d**) using the traditional (Panels **a** & **c**) and fetuses-at-risk (Panels **b** & **d**)approach. Washington State Preterm Births 2002–2011 *n* = 56,537

Under both the traditional and fetuses-at-risk approaches the pattern in the incidence of BPD was very similar for fetuses affected by chorioamnionitis, namely highest in the lowest gestational age ranges (Fig. 2a and b). However, under the traditional model, the incidence of composite BPD and perinatal mortality peaked at early preterm gestations (Fig. 2c), while under the fetuses-at-risk approach, this pattern was only observed for pregnancies affected by chorioamnionitis (Fig. 2d). This differed from the pattern seen for RDS (Fig. 3), whereby the traditional models for both RDS (Fig. 3a) and composite RDS and perinatal death (Fig. 3c) demonstrated a peak in the early preterm period irrespective of the presence of chorioamnionitis and then a steady decline until term gestation. On the other hand, the fetuses-at-risk models (Figs. 3b and d) show a similar pattern with a steady increase throughout the preterm gestation for RDS alone and composite RDS and perinatal death for pregnancies not affected by chorioamnionitis and an early peak with slight decrease for pregnancies complicated by chorioamnionitis.

Using the traditional approach, the crude model showed a statistically significant association between chorioamnionitis and BPD (OR = 5.61, 95% CI: 4.79–6.58) and the composite outcome of BPD and perinatal death (OR = 2.73, 95% CI: 2.42–3.09) (Table 2). Following adjustment for confounders, this relationship was attenuated, but remained statistically significant (BPD aOR = 1.34, 95% CI: 1.09–1.66; BPD or perinatal death aOR = 1.23, 95% CI: 1.01–1.50). Results from the fetuses-at-risk model were similar, albeit much stronger – a significant association was observed between chorioamnionitis and BPD (aOR = 6.25, 95% CI: 5.25–7.44), and between chorioamnionitis and the composite outcome of BPD and perinatal death (aOR = 5.18, 95% CI: 4.39–6.11) following adjustment for relevant confounders (Table 2).

**Table 2:** Logistic regression models examining the association between maternal chorioamnionitis and neonatal respiratory outcomes and perinatal death in preterm infants

	Traditional approach		Fetuses-at-risk approach^b^	
	OR (95% CI)	OR (95% CI)	OR (95% CI)	OR (95% CI)
	BPD	BPD or Perinatal mortality	BPD	BPD or Perinatal mortality
Crude Model	5.61 (4.79–6.58)	2.73 (2.42–3.09)	8.79 (7.53–10.26)	4.22 (3.76–4.73)
Adjusted Model^a^	1.34 (1.09–1.66)	1.23 (1.01–1.50)	6.25 (5.25–7.44)	5.18 (4.39–6.11)
	RDS	RDS or Perinatal Mortality	RDS	RDS or Perinatal Mortality
Crude Model	2.62 (2.39–2.87)	2.45 (2.25–2.68)	3.75 (3.46–4.07)	3.43 (3.19–3.70)
Adjusted Model^a^	0.99 (0.87–1.13)	0.98 (0.86–1.12)	2.78 (2.54–3.06)>	2.74 (2.50–3.01)

^a^Other potential confounders include: diabetes, hypertension, infant sex, maternal age, maternal race, mode of delivery, antenatal steroids. Traditional models are also adjusted for gestational age at delivery

^b^The fetuses-at-risk model included all fetuses-at-risk of respiratory outcomes and perinatal death (*n* = 763,671)

*BPD* bronchopulmonary dysplasia, *RDS* respiratory distress syndrome

The adjusted traditional and fetuses-at-risk models yielded divergent results for RDS (Table 2). Under the traditional approach, adjustment for confounders resulted in non-significant associations between chorioamnionitis and RDS (aOR = 0.99, 95% CI: 0.87–1.13) and between chorioamnionitis and composite RDS and perinatal death (aOR = 0.98, 95% CI: 0.86–1.12). However, under the fetuses-at-risk model the associations between chorioamnionitis and the two respiratory outcomes were significant (RDS OR = 2.78, 95% CI: 2.54–3.06, composite RDS and perinatal death OR = 2.74, 95% CI: 2.50–3.01) (Table 2).

## Discussion

This is the first study examining the association of chorioamnionitis with common neonatal respiratory outcomes using a large administrative population-based database and taking perinatal mortality into account. Using the fetuses-at-risk approach takes all fetuses in utero at a particular gestational age into account, thereby explaining our finding of a slow rise in chorioamnionitis after 30 weeks of gestation, as only a few fetuses are affected at each gestational age. Taking only delivered neonates into account in the denominator, the traditional approach shows a much higher incidence and earlier peak since almost all extremely premature infants are universally affected to some degree by chorioamnionitis [11].

Similarly, in the traditional approach, for both RDS and BPD, both their isolated and composite outcomes combined with perinatal death, are highest in the early gestational ages, irrespective of the presence of chorioamnionitis. However, in the fetuses-at-risk approach the peak in the early gestational ages is largely maintained in the subgroup with chorioamnionitis, suggesting it may itself be associated with preterm delivery. Infants without chorioamnionitis in this model seem to be affected by RDS at later gestational ages which may be accounted for by a higher proportion of infant now born without concerns about chorioamnionitis.

The traditional perinatal model showed that chorioamnionitis appears to be associated with BPD, but not RDS after adjustment for relevant confounders and after accounting for the competing risk of perinatal death. On the other hand, the fetuses-at-risk approach shows a significantly increased risk of BPD and RDS following exposure to chorioamnionitis. Different results using the traditional and fetuses-at-risk approach are not unexpected. The traditional approach models the *prognostic* impact of chorioamnionitis on the development of the fetal lung, while the fetuses-at-risk approach models the *causal* effect of chorioamnionitis on respiratory outcomes. Part of the causality may be the inclination to proceed to delivery earlier with the presence of clinical symptoms of chorioamnionitis. Delivery at lower gestational ages remains the strongest risk factor for development of BPD. The results from this study contradict the early protection, late damage hypothesis, and instead show an increased risk of RDS following chorioamnionitis.

Our results for BPD are congruent with the meta-analysis involving 59 studies and 15,295 infants which showed a significant association between chorioamnionitis and BPD (OR = 1.58, 95% CI: 1.11–2.24) [2]. To our knowledge, only one other study has examined the impact of chorioamnionitis on the composite outcome of BPD or death. This study of 301 infants, which did not adjust for confounders, found a significant crude association between chorioamnionitis and BPD (OR = 3.40, 95% CI: 1.02–11.3) and composite BPD or neonatal death (OR = 2.72, 95% CI: 1.00–7.42) [4]. Comparison of our findings related to RDS with other studies is more difficult as, to our knowledge, no studies have examined the composite outcome of RDS or death. Although one narrative review concluded that chorioamnionitis decreased the incidence of RDS [1], the review did not provide summary statistics. More studies included in the review found a null crude relationship between chorioamnionitis and RDS (6 studies involving 2310 infants) than those that found a significant risk reduction (4 studies involving 1310 infants) [1]. Although adjustment for a variety of confounders showed a negative relationship in some studies, most studies included in the review did not adjust for confounders [1].

Our study has both strengths and limitations. The large population-based sample allowed us to control for multiple potential confounders. Additionally, the use of a recent time period optimizes the applicability of our findings to contemporary neonatal populations. Definitions are based on the most recent consensus document [15], which is particularly relevant for BPD as multiple definitions exist clinically and in the scientific literature [2]. The primary limitations of this study are related to the use of administrative data. Administrative data has not been frequently used to study chorioamnionitis or neonatal respiratory outcomes. A validation study of BPD concluded that ICD-9 codes had sufficient specificity to recommend their routine use to identify this condition [16]. Cases of BPD that were missed tended to be milder in nature [16], which would bias our results towards the null. Similarly, another validation study reported a positive predictive value for 97.0% for the identification of neonatal RDS in administrative data [17], indicating the acceptability of administrative data to identify this condition. To our knowledge, no studies have validated ICD codes for chorioamnionitis. Only one study could be located that used ICD-based codes to identify chorioamnionitis (ICD-9-CM 762.7×, 658.4×) [18]. This population-based study found that chorioamnionitis affected 1.5% of first pregnancies resulting in a live birth and 3.7% of first pregnancies resulting in a stillbirth [18], which is comparable to the overall incidence of 4.65% observed in our study, given that our sample was restricted to preterm births. Our data source did not include information on clinical definitions or the severity of condition and we were unable to differentiate histological and clinical chorioamnionitis. It is estimated that only 8–52% of women with histological chorioamnionitis will display symptoms of clinical chorioamnionitis [10, 19, 20]. However, these results should be interpreted with caution as they come from a selected subsample of placentas that were sent for histological examination and may not reflect the true correlation between clinical and histological chorioamnionitis. Understanding the role of clinical chorioamnionitis which is diagnosed prospectively in the antenatal period (as compared to histological chorioamnionitis which is diagnosed retrospectively after delivery) on infant outcomes is important as it provides clinicians with an opportunity to possibly intervene during pregnancy (using antenatal corticosteroids to promote lung development, or maternal antibiotics). To our knowledge, this is the largest study of the effect of chorioamnionitis on neonatal respiratory outcomes to date. It is also one of very few that accounts for the competing risk of death associated with early chorioamnionitis.

## Conclusions

The fetuses-at-risk analysis used in this study indicates that antenatal inflammation associated with chorioamnionitis leaves the lung vulnerable to RDS and additional post-natal injuries, thus increasing the risk of BPD. Our findings dispute the hypothesis that antenatal inflammation may protect the fetal lung, thus reducing the incidence of RDS. Our study provides evidence confirming that chorioamnionitis increases the risk of RDS, BPD and perinatal death, and pregnancies with chorioamnionitis should be monitored closely and delivered in a facility with appropriate respiratory support and therapy available for infants.

## Abbreviations

BERD: Birth events records database
BPD: Bronchopulmonary dysplasia
CHARS: Comprehensive Hospital Abstract Records Database
CI: Confidence interval
CPAP: Continuous positive airway pressure
ICD-9-CM: International classification of disease version 9 – clinical modification
OR: Odds ratio
RDS: Respiratory distress syndrome
